# Ni mesh-reinforced ultrasonic-assisted Cu/Sn58Bi/Cu joint performance: Experiments and first-principles calculations

**DOI:** 10.1016/j.ultsonch.2024.107119

**Published:** 2024-10-21

**Authors:** Xi Huang, Liang Zhang, Yu-hao Chen, Lei Sun, Xin-quan Yu, Quan-bin Lu

**Affiliations:** a School of Materials Science and Engineering, Xiamen University of Technology, Xiamen 361000, China; b School of Mechanical Engineering and Rail Transit, Changzhou University, Changzhou 213164, China; c State Key Laboratory of Advanced Brazing Filler Metals & Technology, Zhengzhou Research Institute of Mechanical Engineering, Zhengzhou 450001, China

**Keywords:** Ni mesh, Sn58Bi solder, Ultrasonic-assisted soldering, First-principles calculations

## Abstract

The Ni mesh was incorporated into the Cu/Sn58Bi/Cu bonding as a reinforcing skeleton to achieve an enhancement effect analogous to steel reinforcement in concrete. Ultrasonic-assisted soldering (UAS) improved the metallurgical bond among the solder, Ni mesh, and substrate. It facilitated the formation of (Cu, Ni)_6_Sn_5_ intermetallic compounds (IMCs) layers, increasing the joint strength. Observations indicated that ultrasonic treatment effectively refined the (Cu, Ni)_6_Sn_5_ grains and induced a uniform preferred orientation of β-Sn and Bi grains in the joint matrix adjacent to the Ni mesh. The shear strength of the joint reached 72.23 MPa when the ultrasonic application was sustained for 15 s, achieving the fabrication of a high-strength point with low energy consumption. First-principles calculations have confirmed that changes in the Ni content within (Cu, Ni)_6_Sn_5_ IMCs improved the stability of the crystal structure. Furthermore, the variations in content could potentially improve the mechanical and electrical properties of the (Cu, Ni)_6_Sn_5_. Enhancements in ultrasonic efficiency and the reinforcement of IMC structures offer new avenues for research in green and high-performance electronic packaging material joining technologies.

## 1 Introduction

The development of autonomous vehicles signifies a revolutionary advancement in automation and intelligent technology. The innovative technologies underlying this progress, such as precision soldering techniques, have also profoundly impacted the precision manufacturing industry [1], [2]. Within this industry, precision soldering techniques are crucial for ensuring the quality and products performance. The further miniaturization of microelectronic devices and the evolution of integrated circuits towards three-dimensional stacking have set higher standards for joint precision, the enduring stability of connections, and overall performance [3], [4]. These technological innovations necessitate that soldering techniques adapt to finer soldering scales while ensuring the long-term reliability of the solder joints in complex electronic systems. Sn58Bi solder emerges as a preferred lead-free option in precision soldering, offering a low melting point, good wettability, and strong mechanical performance, along with environmental benefits [5]. The UAS technology, with its significant advantages in precision joining, has become a key area of research and application in precision manufacturing. The UAS enhances solder joints by refining microstructures, optimizing thermal distribution, and reducing material deformation and damage. This precise control bolsters mechanical properties at interfaces [6], [7]. Our research on UAS technology aims to enhance joint performance and optimize energy usage, highlighting the importance of dual optimization in current studies.

Li et al. [8] employed UAS technology to regulate the composition of the IMCs at the Ni/Sn/Cu joint interface. When the ultrasonic duration reached 8 s, the joint formed was composed of (Cu, Ni)_6_Sn_5_ IMC, with the grains presenting a uniform circular morphology. The UAS joint exhibited a shear strength of 61.6 MPa, surpassing traditional reflow soldering. Kim et al. [9] successfully fabricated ENIG/Sn-3.5Ag/ENIG solder joints and applied ultrasonication to induce a phase transformation from AuSn_4_ to AuSn_2_, facilitating a reverse peritectic reaction. Solder joints with excellent mechanical properties were successfully obtained in a relatively short bonding time. Wu et al. [10] demonstrated that ultrasound can promote wetting and bonding between SiC ceramics and Zn–Al solder within 20 s. The cavitation effect induced by ultrasound was the primary mechanism for refining metallic bonds in SiC joints. Huang et al. [11] optimized the Cu/SnBi/Cu UAS joint process through orthogonal analysis and experimental validation. The results demonstrated that Cu/Sn58Bi-0.4 Mg/Cu solder joints prepared with “200 ℃, 10 s, 1 kW” exhibited high strength, reaching up to 55.53 MPa. Xu et al. [12] conducted ultrasonic soldering on AA7075 Al alloy using Sn-9Zn solder. They significantly enhanced the acoustic intensity by reducing the joint seam thickness from 0.8 mm to 0.2 mm. Without increasing the ultrasonic energy output, the maximum shear strength of the joint was enhanced to 65.4 MPa, and the hardness was improved to 29.5 HV.

According to the report contributed by Dele-Afolabi et al. [13], interlayer components, as a very promising reinforcing material, feature a three-dimensional network structure that can effectively enhance the interfacial behavior during the diffusion bonding process and optimize thermal conductivity. As an alloying element, Ni has been demonstrated to refine the grain size of Cu_6_Sn_5_ IMC effectively. This research used a high-purity Ni mesh as a reinforcing phase. The introduction of this material was designed to achieve a more uniform reinforcement effect through its regular geometric shape. Furthermore, a Ni mesh with a high specific surface area can potentially increase the solid–liquid interface in the joint, thereby enhancing the soldering efficiency.

This study investigated the microstructure and the formation of IMCs in the Cu/Sn58Bi-Ni mesh/Cu joints under varying ultrasonic application durations (0, 5, 15, 30 s). The joint shear strength, fracture regions, and fracture paths were analyzed to explore how the UAS improved the mechanical properties. In the theoretical investigation, this study employed first-principles analysis to assess the impact of Ni mesh as a reinforcing phase on the joint mechanical and electrical conductivity properties. This research may introduce a new soldering technique to precision manufacturing, enhancing ultrasonic energy efficiency and joint strength.

## 2 Experimental procedures

**Fig. 1:**
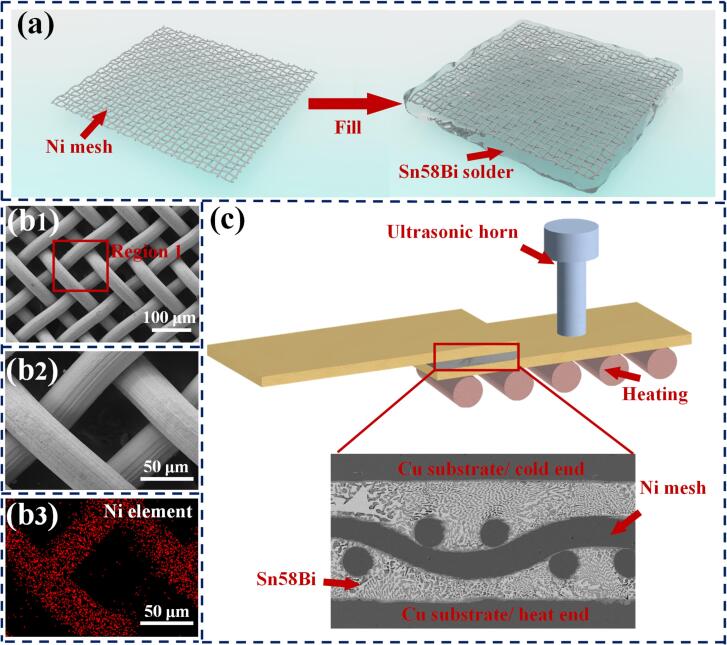
(a) Sn58Bi-Ni mesh preparation, (b_1_)- (b_3_) SEM image of Ni mesh, and (c) schematic diagram of the ultrasonic bonding process.

This study used commercial Sn58Bi solder paste (MLM Material Co., Ltd., China) and high-purity Ni mesh (4 N purity) (Yiminlong Material Co., Ltd., China) to fabricate a composite solder. Fig. 1 (a) shows the Sn58Bi-Ni mesh solder. In an air environment at 30°C, we repeatedly immersed the fluid solder paste into a Ni mesh. Direct pressure of 0.01 MPa was applied to the Sn58Bi-Ni mesh to ensure the expulsion of entrapped air bubbles. The process of immersion and pressing was sustained for 30 min. Fig. 1 (b_1_)- (b_3_) depict the selected #300 Ni mesh, with a pore size of approximately 40 × 40 µm and a wire diameter of about 30 µm. Fig. 1(c) illustrates the bonding process of the Cu/ Sn58Bi-Ni mesh/ Cu joint. Cu substrates, measuring 40 × 4 × 0.5 mm, were utilized as the cold and hot ends of the solder joint. During the bonding process, the joint was heated to 200°C and maintained at this temperature for ultrasonic application durations of 5 s, 15 s, and 30 s, respectively. Throughout the bonding process, a direct pressure of 0.02 MPa was applied to the cold end substrate to stabilize the joint and ensure adequate contact between the molten solder and the substrate. Ultrasonic energy was directly imparted to the hot-end substrate at a frequency of 20 kHz with a power of 200 W. After the ultrasonic application, the joint was allowed to cool naturally to room temperature.

After the bonding process was completed, the cross-section of the joint was first treated with cold embedding using resin. It was then finely ground with SiC sandpaper ranging from #800 to #2500. Finally, the joint was polished to a smooth finish using a cashmere polishing cloth. The 5 % nitric acid-alcohol solution was used to etch the solder joint samples. The microstructure within the joint and the growth of IMCs were examined using Scanning Electron Microscopy (SEM) (Sigma500, Zeiss) and Energy-dispersive X-ray Spectroscopy (EDS) (Max20, Oxford), focusing on the joint elemental distribution. Additionally, the influence of ultrasonic application on the grain growth within the joint was analyzed using Electron Backscatter Diffraction (EBSD) (NordlysMax3, Oxford). The joints were subjected to shear testing in a universal testing machine (DR-509, Dongguan Dongri Co., Ltd., China) at a 2 mm/min strain rate. The dimensions of the lap joint were 4 × 2 mm. The shear strength was obtained from Eq. (1):

(1) τ=F/A

where the shear strength (τ) was obtained by dividing the maximum shear stress (F) by the joint area (A). Each tensile specimen was measured 10 times to obtain the average value and error. The shear strength of the joints was calculated, and the fracture surface morphology and fracture path were examined using SEM.

## 3 Results and discussion

### 3.1 Interface and microstructure

**Fig. 2:**
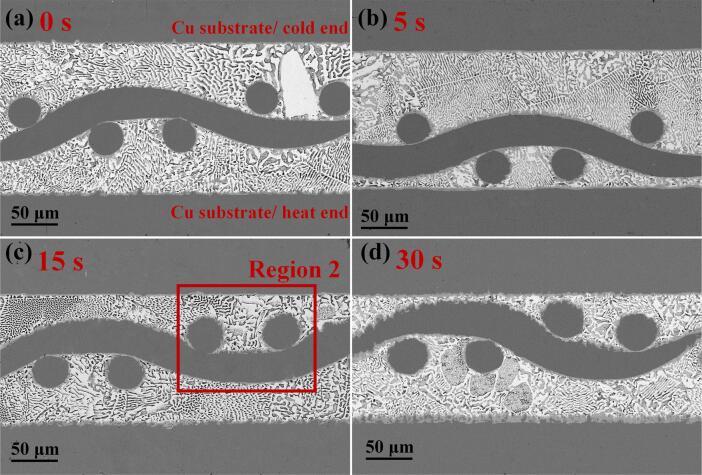
Cu/ Sn58Bi- Ni mesh/ Cu joints with ultrasonic application durations of (a) 0 s, (b) 5 s, (c) 15 s, and (d) 30 s.

**Fig. 3:**
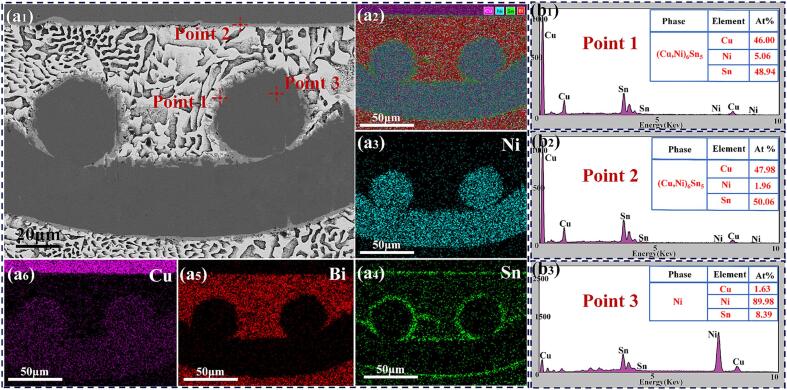
(a_1_)- (a_6_) High-magnification images and elemental analysis of region 2; (b_1_)- (b_3_) EDS results at Points 1, 2, and 3.

Fig. 2 illustrates the interfacial and microstructural growth of the Cu/ Sn58Bi-Ni mesh/ Cu joints under various ultrasonic application durations. The width of the joint was approximately 140 μm, with the Sn58Bi solder completely filling the mesh apertures of the Ni mesh. The Ni mesh maintained a three-dimensional continuous and regular mesh structure, which endowed the joint with an interpenetrating reinforcement structure. EDS results shown in Fig. 3(b_1_) indicated the formation of (Cu, Ni)_6_Sn_5_ IMC at the Ni mesh /solder matrix interface. This phenomenon was attributed to the close atomic radii of Ni and Cu atoms (1.28 Å and 1.25 Å, respectively), which enabled Ni atoms to substitute for some Cu atoms, forming a substitutional solid solution:

(2) $$6\left(1-x\right)Cu+5Sn+6xNi\to {\left(Cu_{1-x}Ni_{x}\right)}_{6}Sn_{5}+\Delta H$$

This demonstrated that an effective metallurgical bond was established between the Ni mesh and the solder matrix, significantly enhancing the interfacial bond strength. Additionally, based on the EDS results shown in Fig. 3(b_2_), the formation of a (Cu, Ni)_6_Sn_5_ IMC layer was also observed at the solder /Cu substrate interface. However, the Ni content in the IMC layer at the substrate was lower than at the Ni surface. As shown in Fig. 3(a_6_), the distribution of Cu atoms in the joint did not exhibit vertical uniformity from high to low concentration; instead, an attractive interaction with Ni atoms was observed. Wang et al. [14] reported that at the Sn/Ni interface, the mixing of Cu and Ni atoms within the Cu-Sn-Ni ternary phase led to an increase in entropy, which in turn drove the dissolution and accumulation of the incoming Cu atoms in the ternary phase.

After ultrasonication was applied, Fig. 2(a) indicated that the coarsening phenomenon of Bi had been mitigated. This effect was attributed to the high-intensity ultrasonic injection, which enhanced the melt homogeneity and promoted the fragmentation of the solid phase, thereby achieving a refinement effect on dendrites or the solid phase during the crystallization nucleation process [15]. Table 1 displays the variation in the thickness of the IMC layer on Cu substrates. The thickness of the IMC layer was determined by dividing the area of the IMC, as measured using ImageJ software, by its corresponding length. As the ultrasonic duration increased, the IMC layer thickened due to the ultrasonic wave, promoting Cu, Ni, and Sn diffusion. Gao et al. [16] calculated the transport driving force ΔG for Sn across the interface between the solder and the IMC region. They found that the absolute value of ΔG decreased monotonically with increasing Ni concentration. This finding indicated that an increase in Ni atom concentration enhanced the growth of the IMC region. The formation enthalpy of (Cu, Ni)_6_Sn_5_ calculated in the following sections can also account for the improved growth of the IMC. Due to the continuous action of cavitation bubble implosions, when the ultrasonic duration reached 30 s, partial dissolution occurred on the surface of the Ni mesh. Concurrently, etch pits formed on the surface of the Cu substrate, as shown in Fig. 2(d). The occurrence was attributed to the implosion of cavitation bubbles that generated strong shock waves, with the transient temperature of the surrounding liquid peaking at 1600 ℃ [17]. The Cu substrate was likely subject to localized melting or softening, culminating in the formation of pits from the impact of these shock waves. Additionally, the IMC layer was observed to have provided new diffusion paths for Cu and Sn atoms, and the confluence of chemical gradients and ultrasonic streaming was found to have facilitated the more effective diffusion of Cu and Ni atoms into the matrix. The uneven interface facilitated more thorough contact between the solder and substrate material, thereby increasing the bonding area, which was conducive to enhancing the shear stress resistance of the joint. Moreover, the fine IMC structures fragmented by ultrasonic vibration can provide heterogeneous nucleation sites, further strengthening the mechanical properties of the joint.

**Table 1:** IMC thickness of solder joints on Cu substrates.

UAS duration (s)	Cold end IMC thickness(μm)	Hot end IMC thickness(μm)
0	1.53	1.77
5	2.43	3.98
15	3.32	6.12
30	4.89	9.73

### 3.2 EBSD analysis of the joint

**Fig. 4:**
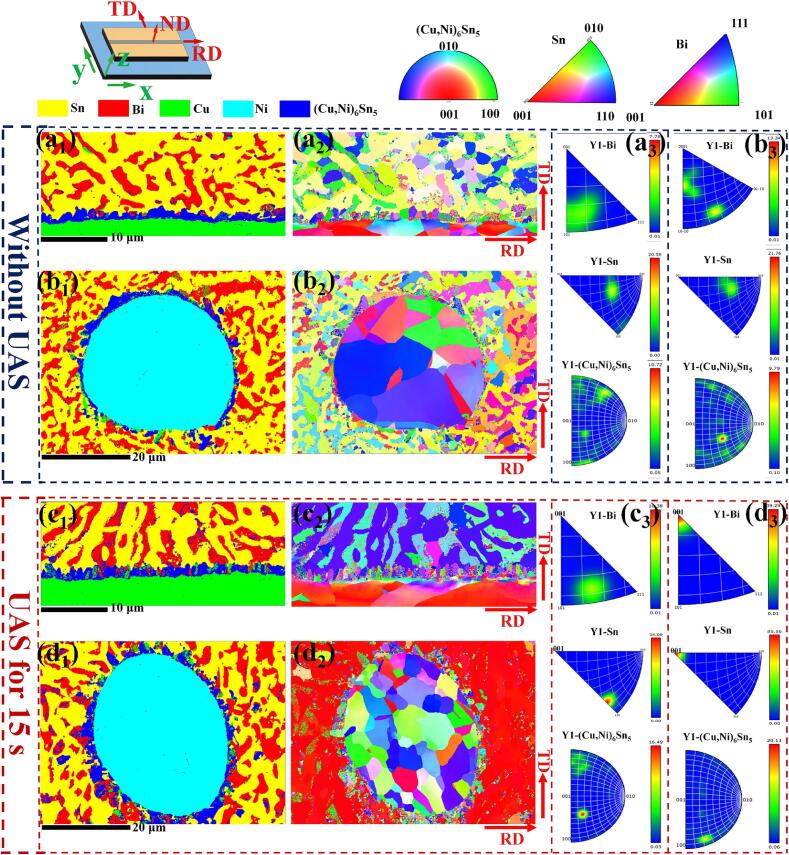
(a_1_)- (d_1_) Phase distribution map; (a_2_)- (d_2_) IPFs on Y-axis; (a_3_)- (d_3_) The antipode diagram on Y-axis.

To further investigate the impact of ultrasonic treatment on the grain growth within the joint, EBSD was employed to analyze the joint microstructure. Fig. 4 presents the phase distribution map and the inverse pole figures (IPFs) along the Y-axis direction (i.e., the soldering direction). From the phase distribution map, the (Cu, Ni)_6_Sn_5_ IMC layer on the Ni mesh surface changed from discontinuous to continuous after applying ultrasound. Moreover, there were more free IMC fragments in the matrix. Upon analysis of Fig. 4(a_2_)–(b_2_), it was observed that the β-Sn and Bi grains exhibited random crystallographic growth orientations without ultrasonic application. The grain size of the (Cu, Ni)_6_Sn_5_ was relatively large, presenting a coarser morphology. After the application of ultrasonication, the morphology of the (Cu, Ni)_6_Sn_5_ grains on the substrate side became elongated and maintained a random crystallographic orientation. In the regions adjacent to the Ni mesh, β-Sn and Bi grains exhibited a pronounced and preferred orientation along the (001) crystallographic direction, as demonstrated in Fig. 4(d_2_). This phenomenon was also confirmed according to the antipode diagram in Fig. 4(d_3_). The grain sizes of the (Cu, Ni)_6_Sn_5_ on the Cu substrate side and at the Ni mesh surface were determined, which were found to be 0.97 μm and 0.6 μm in the absence of ultrasonic treatment, and 0.82 μm and 0.45 μm after the UAS, respectively. Although the thickness of the IMC layer has increased, the refinement of grains continued to contribute significantly to the material strength, as demonstrated in previous studies [18]. Grain refinement was due to ultrasonic cavitation, which enhanced nucleation and increased the solder undercooling, leading to finer grains. Furthermore, Ni addition promoted dispersed nucleation of IMC grains, increasing nucleation density and refining grain size. The UAS can enhance the formation of uniform preferred orientations in β-Sn and Bi phases within the joint matrix. Similarly, Chen et al. [19] have successfully transitioned the orientation of β-Sn and Bi grains in the matrix from random to preferred orientation using the UAS. Additionally, the inhomogeneous distribution of local temperature and stress fields within the joint may account for the observed differences in crystal growth kinetics, a topic discussed in existing literature [20], [21]. This phenomenon opens a new research path to explore the mechanism of ultrasound-induced optimal orientation of solder joints.

### 3.3 Mechanical properties

**Fig. 5:**
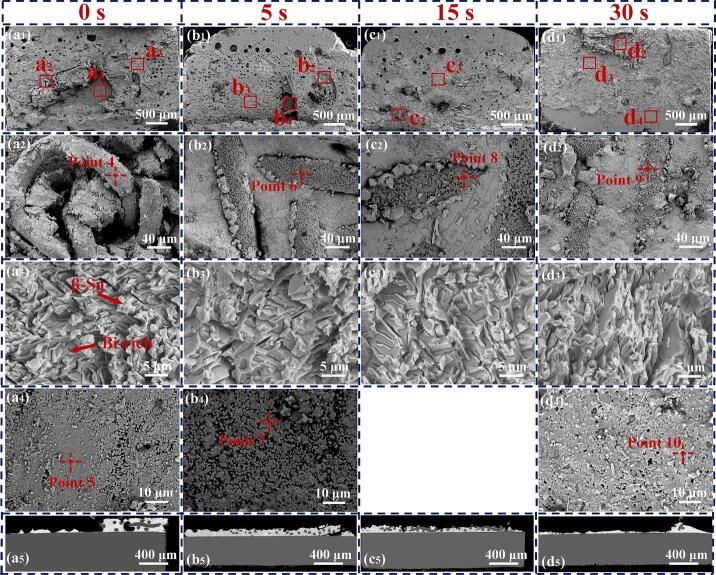
(a_1_) − (d_1_) Macroscopic overview of the fracture surface of the joint. Characteristics of (a_2_) − (d_2_) Ni mesh surface, (a_3_) − (d_3_) solder matrix, and (a_4_) − (d_4_) (Cu, Ni)_6_Sn_5_ IMC layer on the Cu substrate side at the fracture. (a_5_) − (d_5_) Fracture cross-sectional view.

SEM images of the joint fracture surfaces, resulting from UAS at different durations (0, 5, 15, 30 s) are presented in Fig. 5(a_1_)–(d_1_). All specimens showed fractures occurring at the joint, with the displayed fracture surfaces on the hot end. Fig. 5(a_2_)–(d_2_) display magnified images of the fractures within the Ni mesh. In Fig. 5(a_2_), the structural integrity of the Ni mesh was compromised during the shear process of the joint. The Ni mesh surface displayed an absence of a fully formed IMC layer. These observations are consistent with the phase distribution results obtained from EBSD results in the preceding section. In contrast, following ultrasonic treatment, the integrity of the Ni mesh structure within the fracture region was preserved, and the Ni mesh surface was entirely enveloped by a compact layer of (Cu, Ni)_6_Sn_5_ grains. The elemental results are presented in Table 2, and the EDS results from points 4, 6, 8, and 9 will serve as the basis for modeling in DFT calculations. The data presented in Table 2 showed that the relative proportion of Ni atoms in (Cu, Ni)_6_Sn_5_ grains increased with the prolongation of ultrasonic duration. This increase was likely facilitated by the cavitation effect, which promoted the interdiffusion of atoms. Fig. 5(a_3_)–(d_3_) depicte the microstructure of the solder matrix within the joint fracture region, predominantly consisting of β-Sn and a Bi-rich phase. The fracture characteristics were indicative of semi-ductile fracture behavior. Such a fracture pattern is commonly observed in joints with enhanced mechanical strength. Fig. 5(a_4_)–(d_4_) depicted the regions of fracture within the (Cu, Ni)_6_Sn_5_ IMC layer on the Cu substrate. Upon the 15 s ultrasonic treatment, fractures were not observed within the (Cu, Ni)_6_Sn_5_ IMC layer on the Cu substrate surface. Additionally, the Ni content within the (Cu, Ni)_6_Sn_5_ at the substrate increased with the prolongation of ultrasonic treatment duration. It was consistently lower than that on the Ni mesh surface, corroborating the previous EDS results. Fig. 5(a_5_)–(d_5_) presents the fracture cross-sectional views under various ultrasonic treatment durations. These images intuitively show a reduction in the brittle fracture phenomena at the (Cu, Ni)_6_Sn_5_ interface following ultrasonic treatment. However, at an ultrasonic treatment duration of 30 s, fractures were more frequently observed at the Cu substrate side of the (Cu, Ni)_6_Sn_5_ interface.

**Table 2:** EDS results from the solder joint.

	Cu (in at%)	Ni (in at%)	Sn (in at%)	Measured (Cu, Ni): Sn	Theoretical (Cu, Ni): Sn
Point 4	46.33	4.75	48.92	51.08: 48.92	6:5
Point 5	56.85	2.17	40.98	59.02: 40.98	6:5
Point 6	43.58	9.12	47.30	52.7: 47.30	6:5
Point 7	52.43	4.26	43.31	56.69: 43.31	6:5
Point 8	44.4	13.88	41.72	58.28: 41.72	6:5
Point 9	40.79	18.43	40.78	59.22: 40.78	6:5
Point 10	43.83	7.25	48.93	51.08: 48.93	6:5

**Fig. 6:**
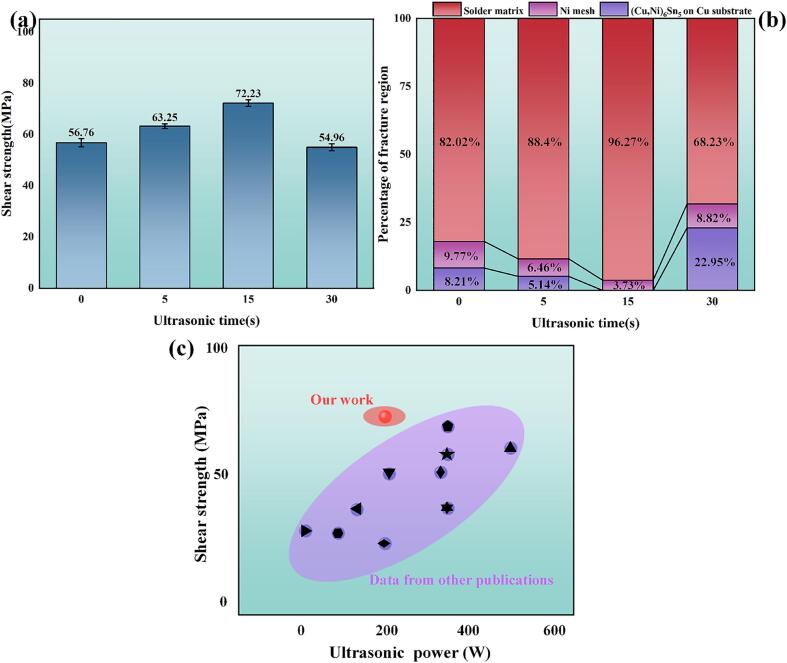
(a) Joint shear strength at various ultrasonic treatment durations, (b) proportional distribution of fracture area types, and (c) comparison between the current experimental results and those reported in the literature: [23], [24], [25], [26], [27], [11], [28], [22], [29], [30].

Fig. 6(a) and (b) depicte the influence of ultrasonic treatment duration on the joint shear strength, which was enhanced as the ultrasonic application time increased. Fractures in the joints predominantly occurred within the solder matrix. Notably, at an ultrasonic duration of 15 s, the shear strength was maximized, reaching 72.23 MPa, with fractures within the solder matrix being most prevalent and minimal on the Ni mesh surface. However, when the ultrasonic duration was extended to 30 s, the shear strength was reduced, falling below the levels of joint without ultrasonic treatment. This decrease was likely associated with the excessive and irregular growth of the IMC layer, potentially leading to crack propagation and a mismatch in the coefficient of thermal expansion (CTE). Hu et al. [22] reported that the interlocking reinforcement structure of metal foam effectively impeded the propagation of micro-cracks, leading to an enhancement in shear strength. The grain refinement caused by cavitation effects significantly enhanced the strength of the solder joint. Additionally, the UAS increased Ni concentration in (Cu, Ni)_6_Sn_5_, enhancing mechanical properties, as supported by further DFT calculations.

A selection of literature on ultrasonic-assisted Cu-Cu bonding was examined, focusing on the correlation between ultrasonic power output and the resultant shear strength of the joints. A comparison of the shear strength of ultrasound-assisted Cu-Cu joints in this and other reported experiments is shown in Fig. 6(c). It was clearly observed that the Cu/ Sn58Bi-Ni mesh/ Cu joint exhibited higher shear strength under lower ultrasonic power. This phenomenon was confirmed to enhance the joint strength by the Ni mesh as a reinforcing material, and it also led to a significant improvement in energy utilization efficiency.

### 3.4 Mechanism of Ni mesh-enhanced cavitation effect

Ultrasound waves induce acoustic cavitation effects as they propagate through a liquid medium. Upon nearing the solid–liquid interface, the cavitation bubbles encounter a restriction in their movement, resulting in their abrupt and localized implosion at the interfacial region. Localized micro-jets are generated within the contact region between the solid and the liquid [31]. The effective area of micro-jet occurrence is determined by the diameter of the cavitation bubbles, with the zone of maximum influence typically being twice the diameter of the cavitation bubbles [32]. To analyze “ultrasonic utilization efficiency,” the assumption that the diameter of the cavitation bubbles is approximately 20 μm has been adopted, as proposed by Xu et al. [33].

**Fig. 7:**
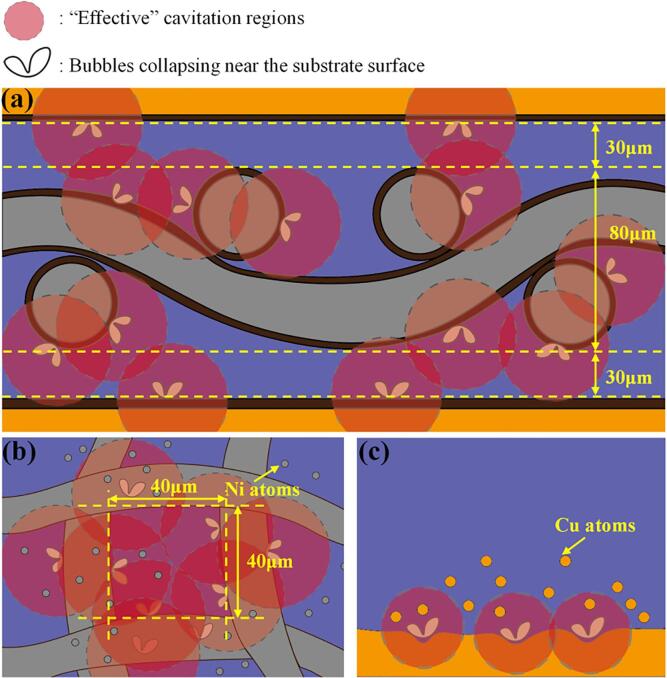
Cavitation effect schematic diagram: (a) Entire solder joint, (b) Ni mesh area, and (c) Cu substrate side.

Fig. 7(a) illustrates a schematic of cavitation effects occurring within the joint. The joint height measured 140 μm, as determined in the previous section. The Ni mesh thickness within the joint cross-section was 80 μm, with 30 μm spacing above and below the mesh to the substrate. It was found that the effective cavitation zone produced by cavitation bubbles on the Cu substrate and Ni mesh surfaces encompassed the entire joint. Fig. 7(b) illustrates a schematic of the cavitation effect within the Ni mesh apertures, where the 40 μm aperture diameter was comparable to the effective zone of individual cavitation bubbles, indicating significant cavitation effects had occurred internally. Fig. 7(c) presents a schematic illustration of the cavitation effect on the Cu substrate. Some Cu atoms were involved in forming the IMC, while the rest were diffused into the solder matrix due to the temperature gradient. Overall, Ni mesh has led to a focused distribution of ultrasonic energy, substantially increasing the efficiency of ultrasonic energy utilization. This advancement enhances the energy utilization efficiency of ultrasonic techniques and introduces innovative approaches toward achieving green and sustainable industrial practices.

## 4 First-Principles Calculation

### 4.1 Preferred occupations of Ni atoms and lattice parameter

First-principles calculations of (Cu, Ni)_6_Sn_5_ IMCs were performed using DFT with the Cambridge sequential total energy package (CASTEP) code. According to previous reports [34], two primary forms of the Cu_6_Sn_5_ phase have been identified: η-Cu_6_Sn_5_ and η'-Cu6Sn_5_. η-Cu_6_Sn_5_ (hexagonal) forms at high temperatures and transforms into the more stable η'-Cu_6_Sn_5_ (monoclinic) structure when the temperature drops below 186°C [35]. The η'-Cu6Sn5 phase was selected as the initial structure for this experiment. (Cu, Ni)_6_Sn_5_ is a substitutional solid solution formed by doping Ni atoms and replacing Cu atoms in Cu_6_Sn_5_. Based on the EDS results from the Points (4, 6, 8, 9) in the previous section, we constructed four structural models: (Cu_5.5_, Ni_0.5_)Sn_5_, (Cu_5_, Ni_1_)Sn_5_, (Cu_4.5_, Ni_1.5_)Sn_5_, and (Cu_4_, Ni_2_)Sn_5_. The minimum formation enthalpy for the four configurations was determined using Eq. (3). The lattice parameters and internal atomic coordinates were optimized based on the principle of energy minimization to identify the most stable structure.

(3) $$E_{f}=\left[E_{\mathrm{IMCs}}^{\mathrm{total}}-\left(m\mu_{\mathrm{Cu}}+n\mu_{\mathrm{Ni}}+k\mu_{\mathrm{Sn}}\right)\right]/44$$

$E_{\mathrm{IMCs}}^{\mathrm{total}}$ represents the total energy of the doped IMC, while $m\mu_{\mathrm{Cu}}$, $n\mu_{\mathrm{Ni}}$, and $k\mu_{\mathrm{Sn}}$ denote the total chemical formulas of Cu, Ni, and Sn within the unit cell, respectively. 44 represents the total number of atoms in the doping system.

**Fig. 8:**
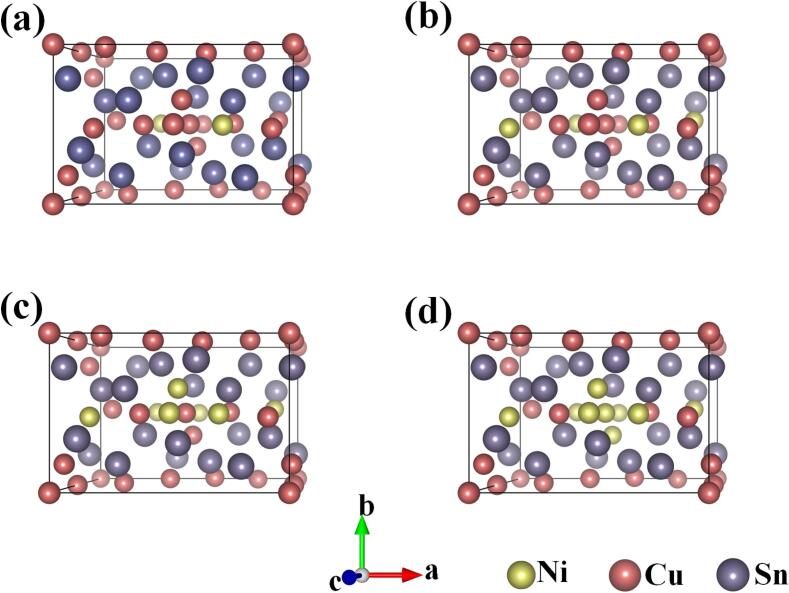
Doped system crystal structures of (a) −2Ni, (b) −4Ni, (c) −6Ni, and (d) −8Ni.

Table 3 presents the optimized crystallographic parameters and the formation enthalpy per atom. Fig. 8 depicts the corresponding crystal structures. It was observed from Table 3 that adding Ni reduced the formation enthalpy of IMCs. Lower formation enthalpy typically indicated a more favorable energy state for stable crystal structures. In the following text, (Cu_24-_*_x_*, Ni*_x_*)Sn_20_ is denoted as −*x*Ni, (*x* = 2, 4, 6, 8).

**Table 3:** Lattice parameters and formation enthalpy of the doped system.

IMCs	Lattice parameter						*H_f_* (meV/atom)
	*a*(Å)	*b*(Å)	*c*(Å)	*α*(°)	*β*(°)	*γ*(°)	
−2Ni	10.973	7.332	10.100	90.039	98.637	90.153	−3.712
−4Ni	11.014	7.292	9.979	89.805	99.075	89.854	−3.750
−6Ni	11.026	7.283	9.929	90.055	99.506	89.596	−3.788
−8Ni	11.028	7.275	9.861	90.095	99.702	89.531	−3.826

### 4.2 Elastic moduli of IMCs

The response of materials to external stress is reflected in their elastic properties. The elastic modulus is influenced by the strength of the chemical bonds within the material and is closely associated with its mechanical properties and dynamic characteristics [36]. In the previous section, four configurations of (Cu, Ni)_6_Sn_5_ were obtained. The elastic constants of the various doped systems were calculated using the stress–strain method. According to the report by Xu et al. [37], the 13 independent elastic constants (C_ij_) for the monoclinic system were identified. The calculated C_ij_ for the four different configurations constructed in this study are shown in Table 4. The Eq. (4) serves as the criterion for determining the structural stability of monoclinic systems. Data verification confirms that all four doped structures exhibit excellent mechanical stability. Furthermore, all doped systems satisfied the “Cauchy pressure criterion [38]” for C_ij_ (C_12_-C_44_ > 0), thereby exhibiting good ductility.

(4) $$\begin{array}{c} C_{\mathrm{ii}}>0,\left[C_{11}+C_{22}+C_{33}+2\left(C_{12}+C_{13}+C_{23}\right)\right]>0,\\ C_{33}C_{55}-C_{35}^{2}>0,C_{44}C_{66}-C_{46}^{2}>0,C_{33}C_{22}-2C_{23}>0 \end{array}$$

**Table 4:** Stiffness coefficients C_ij_ for doping systems.

IMCs	−2Ni	−4Ni	−6Ni	−8Ni
C_11_	149.02602	146.39731	145.37598	147.60288
C_22_	132.62826	138.58776	139.29102	135.31848
C_33_	110.80469	128.88571	125.97395	128.22274
C_44_	40.14112	36.52881	31.91886	35.76405
C_55_	51.83336	53.71786	46.44286	46.9829
C_66_	45.65781	42.70614	33.39317	33.11217
C_12_	45.43051	51.26642	54.42944	55.40677
C_13_	48.66238	53.2455	58.87821	62.00746
C_15_	1.68317	2.68546	−3.13885	−3.14173
C_23_	42.28811	44.73864	51.05864	64.11392
C_25_	5.39689	6.40368	8.12304	10.72061
C_35_	−6.57738	−1.89544	−3.23888	−3.04223
C_46_	1.83276	2.27395	1.62024	1.37113

**Fig. 9:**
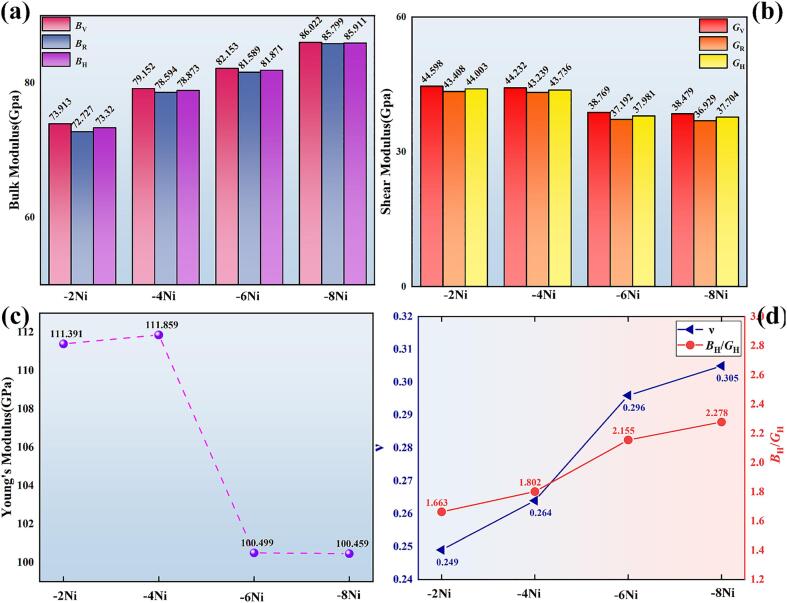
The (a) *B*, (b) *G*, (c) *E*, (d)*v* and *B*_H_/*G*_H_ of the dopant structure.

The *B* (bulk modulus) measures a material ability to change volume under uniform pressure. The *G* (shear modulus) assesses deformability under shear stress. The *E* (Young's modulus) is used to evaluate the ability to deform under axial tension or compression. The elastic moduli of different doping systems were approximated using the Voigt-Reuss-Hill method [37], as shown in Fig. 9. The *B*, *G*, *E*, and *ν* (Poisson's ratio) of the polycrystalline materials were calculated using Eq. (5):

(5) $$\begin{array}{c} G=\left(G_{V}+G_{R}\right)/2,B=\left(B_{V}+B_{R}\right)/2\\ E=9GB/\left(G+3B\right),\upsilon =\left(3B-E\right)/6G \end{array}$$

where, *G_V_* and *B_V_* were determined using the Voigt method, and *G_R_* and *B_R_* were determined using the Reuss method. According to the computational results shown in Fig. 9(a), the increase in Ni doping content led to a rising trend in the *B* of the system. This indicated a significant enhancement in the resistance of the IMC to external compression within the elastic regime. When the doping content reached 8 Ni atoms, the *B* of the IMCs reached its maximum value of 86.022 GPa. The critical value of the *B* to *G* ratio (*B*_H_/*G*_H_) that distinguishes brittle from ductile materials was approximately 1.75 [39]. The incorporation of Ni resulted in the IMCs progressively exhibiting more pronounced ductility. Furthermore, by comparing *E*, −2Ni and −4Ni IMCs exhibit higher hardness than −6Ni and −8Ni IMCs. When the *ν* exceeds 0.26, the material demonstrates ductility; below this threshold, it exhibits brittleness [40]. Doping a single crystal with two Ni atoms rendered the IMCs brittle, but as the Ni atoms doped increased, the IMCs progressively exhibited more pronounced ductility. This is consistent with the conclusions drawn from the Cauchy stress analysis. Overall, increasing the amount of Ni doping effectively enhances the shear stability and ductility of IMCs.

**Fig. 10:**
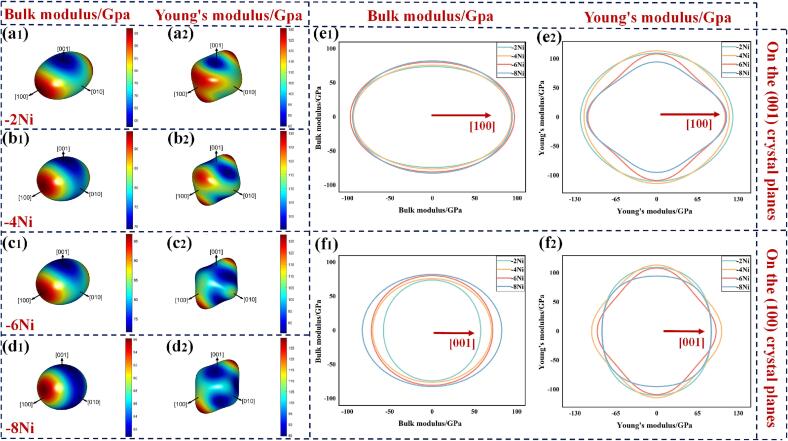
(a_1_)- (d_1_) The *B* and (a_2_)- (d_2_) *E* on a three-dimensional map. Projections of *B* and *E* on (e_1_)- (e_2_) (001) and (f_1_)- (f_2_) (100) crystal planes.

Investigating material anisotropy is crucial as it unveils the underlying reasons for performance variations across different directions. The spatial distribution of the *B* and the *E* on the three-dimensional surfaces of a monoclinic crystal system is simulated using Eqs. (6), (7):

(6) $$\begin{array}{c} \frac{1}{B}=\left(S_{11}+S_{12}+S_{13}\right)l_{1}^{2}+\left(S_{12}+S_{22}+S_{23}\right)l_{2}^{2}+\left(S_{13}+S_{23}+S_{33}\right)l_{3}^{2}\\ +\left(S_{15}+S_{25}+S_{35}\right)l_{1}l_{3} \end{array}$$

(7) $$\begin{array}{c} \frac{1}{E}=l_{1}^{4}S_{11}+2l_{1}^{2}l_{2}^{2}S_{12}+2l_{1}^{2}l_{3}^{2}S_{13}+2l_{1}^{3}l_{3}S_{15}+l_{2}^{4}S_{22}+2l_{2}^{2}l_{3}^{2}S_{23}+2l_{1}l_{2}^{2}l_{3}S_{25}+l_{3}^{4}S_{33}\\ +2l_{1}l_{3}^{3}S_{35}+l_{2}^{2}l_{3}^{2}S_{44}+2l_{1}l_{2}^{2}l_{3}S_{46}+l_{1}^{2}l_{3}^{2}S_{55}+l_{1}^{2}l_{2}^{2}S_{66} \end{array}$$

where, *S*_ij_ (elastic flexibility constants) and *C*_ij_ are inverse matrices of each other. *l*_1_, *l*_2_, and *l*_3_ denote direction cosines relative to the *x* , *y*, and *z* axes, respectively. Fig. 10(a_1_)−(d_1_) illustrate the anisotropy of the bulk modulus in the IMCs crystals for four different doped systems in a 3D model, while Fig. 10(a_2_)−(d_2_) show the anisotropy of the *E*. Compared to *B*, the 3D structure of *E* in IMCs was observed to deviate more significantly from a spherical shape. This indicated that the anisotropy of *E* was more pronounced in the doped systems, making *E* more independent of the crystallographic direction. From Fig. 10(e_1_)−(f_1_), it can be seen that the anisotropy of the *B* in IMCs is most pronounced on the (001) crystallographic plane, particularly along the [100] direction. On the (100) crystallographic plane, it was observed that the *B* of IMCs increased in all directions, with the fastest increase along the [001] direction. The projection changed from a uniform circle to an ellipse. With the increase in Ni atoms, the anisotropy of the *E* in IMCs became more pronounced along the [100], [001], and [010] crystallographic directions. However, when more than four Ni atoms were doped, there was a trend in IMCs where the *E* transitioned from increasing to decreasing. Similarly, Jiang et al. [41] observed using micropillar compression techniques that the *E* of Cu_6_Sn_5_ decreased by 7 % as the crystal grains shifted from near the [001] direction to perpendicular to it. Luktuke et al. [42] found that adding indium reduced the *E* of Cu_6_Sn_5_ regardless of crystal orientation and indium atom position. The observed decrease in shear strength of solder joints in this experiment was hypothesized to be related to the decrease in the *E* of IMCs.

### 4.3 Electronic structure

**Fig. 11:**
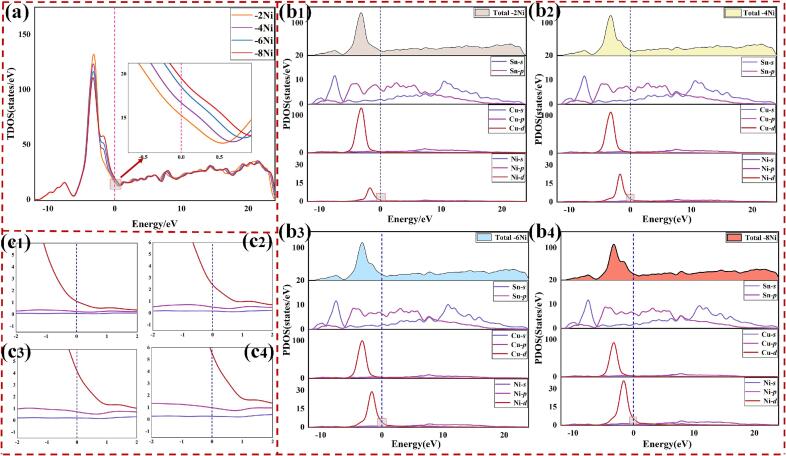
(a) PDOS of doped structure; TDOS of (b_1_) −2Ni IMC, (b_2_) −4Ni IMC, (b_3_) −6Ni IMC, (b_4_) −8Ni IMC; (c_1_) − (c_4_) are the amplified data regions of (b_1_) − (b_4_).

An analysis of the electronic structure's density of states was performed for doped IMC configurations to elucidate the composition of IMC stability. Fig. 11(a) depicts the Total Density of States (TDOS) for four configurations, while Fig. 11(b) presents the Partial Density of States (PDOS). A binding peak was observed at −3.11 eV, primarily contributed to by Cu-*d* and Sn-*p* states in the density of states. Near the Fermi level, a pseudogap was observed in the energy range of −2.0 to −1.5 eV. The Fermi level was located approximately 0.5 eV above the pseudogap, indicating the stable doped structure [43]. This is consistent with conclusions that were drawn from the *H*_f_ analysis.

In addition, the density of states near the Fermi level was increased with doping Ni, indicating enhanced electrical conductivity of IMCs. Conversely, the increased density of states near the Fermi level suggested a decrease in the structural stability of IMCs, which was correlated with the reduction of *E*. The Ni-*d* orbitals were closer to the Fermi level, as shown in Fig. 11(c). The TDOS of Ni-*d* orbitals near the Fermi level continued to increase, further revealing Ni atoms enhancement of metallic properties in IMCs.

**Fig. 12:**
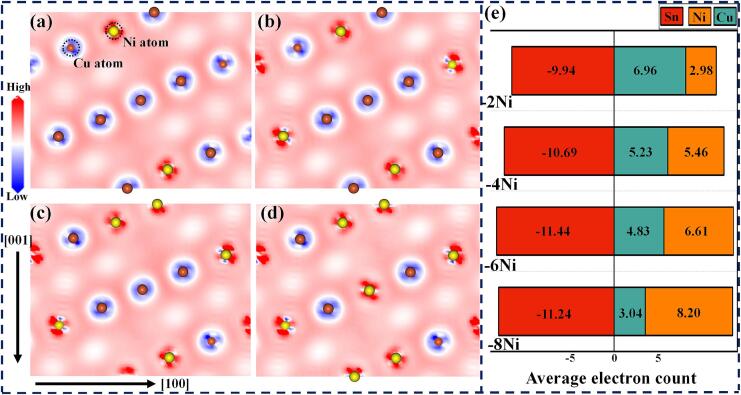
The charge density difference on the (010) plane of (a) −2Ni IMC, (b) −4Ni IMC, (c) −6Ni IMC, and(d) −8Ni IMC. (e) Bader charge transfer analysis.

The charge density distribution of doped systems was computed to investigate atomic bonding strength further. Fig. 12(a)−(d) illustrate the charge density distribution along the (010) crystal plane for four doping configurations. With an increase in the number of Ni atoms, a significant enhancement in internal charge exchange was observed, thereby increasing binding energy. Additionally, Fig. 12(e) documents the average electron transfer for the atoms. Sn atoms lost electrons during the electron exchange, while Cu and Ni atoms gained electrons. Based on the electron exchange of Sn atoms, the total electron exchange amounts for the four doping systems were determined to be 9.94, 10.69, 11.44, and 11.24, respectively. This result confirmed that charge redistribution was enhanced with the increasing number of Ni atoms. Specifically, in the −6Ni IMC, electron transfer was more intense, indicating more stable atomic bonding strength. This could explain why the −6Ni IMC on the Ni mesh surface in the solder joint was less prone to fracture. The results indicate that Ni doping has altered the electronic structure of the IMCs, potentially enhancing their electrical conductivity and bonding strength.

## 5 Conclusions

(1) Through the UAS, the Ni mesh successfully formed a reliable metallurgical bond with the solder matrix, forming (Cu, Ni)_6_Sn_5_ IMC layers on the Ni mesh surface and the Cu substrate side. The refinement of IMC grains was facilitated by the ultrasonic cavitation effect and nucleation catalysis of Ni. Furthermore, in the matrix near the Ni mesh, β-Sn and Bi grains exhibit a preferred orientation with the (001) direction parallel to the soldering direction.
(2) After ultrasonication, most of the fracture sites in the joint occurred within the solder matrix, enhancing the joint shear strength. When the UAS was applied for 15 s, the joint shear strength notably increased to 72.23 MPa. Compared to the published reports, a higher shear strength was achieved with relatively lower ultrasonic power.
(3) Employing first-principles calculations, we conducted an in-depth analysis of the effects of varying Ni content in (Cu, Ni)_6_Sn_5_ IMC on the elastic modulus and electrical properties. Theoretical insights have revealed that increasing Ni content within (Cu, Ni)_6_Sn_5_, induced by ultrasonic treatment, is crucial in enhancing joint strength.

## CRediT authorship contribution statement

**Xi Huang:** Writing – review & editing, Writing – original draft, Supervision, Software, Resources, Project administration, Methodology, Investigation, Funding acquisition, Formal analysis. **Liang Zhang:** Data curation, Conceptualization. **Yu-hao Chen:** Visualization, Validation, Supervision. **Lei Sun:** Software, Resources, Project administration. **Xin-quan Yu:** Methodology. **Quan-bin Lu:** Visualization, Validation.

## Declaration of competing interest

The authors declare that they have no known competing financial interests or personal relationships that could have appeared to influence the work reported in this paper.
